# Predictive Efficacy of Dual Therapies Combining Integrase Strand Transfer Inhibitors with Second-Generation Non-Nucleoside Reverse Transcriptase Inhibitors Following HIV-1 Treatment Failure in Cameroon: Implications for the Use of a Long-Acting Therapeutic Strategy in Low- and Middle-Income Countries

**DOI:** 10.3390/v16121853

**Published:** 2024-11-29

**Authors:** Davy-Hyacinthe Gouissi Anguechia, Yagai Bouba, Ezechiel Ngoufack Jagni Semengue, Aude Christelle Ka’e, Désiré Takou, Collins Ambe Chenwi, Grace Beloumou, Alex Durand Nka, Ulrich Roland Basseck Wome, Maria Mercedes Santoro, Francesca Ceccherini-Silberstein, Adawaye Chatté, Carla Montesano, Giulia Cappelli, Vittorio Colizzi, Alexis Ndjolo, Dora Mbanya, Nicaise Ndembi, Carlo-Federico Perno, Joseph Fokam

**Affiliations:** 1 Chantal BIYA International Reference Centre for Research on HIV/AIDS Prevention and Management, Yaoundé P.O. Box 3077, Cameroon; davygouissi@gmail.com (D.-H.G.A.); romeobouba@yahoo.fr (Y.B.); ezechiel.semengue@gmail.com (E.N.J.S.); kae.audechristelle@gmail.com (A.C.K.); dtakou@yahoo.com (D.T.); collinschen@yahoo.co.uk (C.A.C.); graceangong12@yahoo.fr (G.B.); nkaalexdurand@yahoo.com (A.D.N.); freedywilliam@gmail.com (U.R.B.W.); colizzi@bio.uniroma2.it (V.C.); andjolo@yahoo.com (A.N.); carlofederico.perno@opbg.net (C.-F.P.); 2 Faculty of Medicine and Biomedical Sciences, University of Yaoundé I, Yaoundé P.O. Box 337, Cameroon; dmbanya1@yahoo.co.uk; 3 Faculty of Medicine, UniCamillus-Saint Camillus International University of Health Sciences, 00131 Rome, Italy; 4 National HIV Drug Resistance Working Group, Yaoundé P.O. Box 1459, Cameroon; 5 Department of Experimental Medicine, University of Rome “Tor Vergata”, 00133 Rome, Italy; santormaria@gmail.com (M.M.S.); ceccherini@med.uniroma2.it (F.C.-S.); 6 Project Management Unit, Ministry of Health, N’djamena P.O. Box 548, Chad; cadawaye@yahoo.fr; 7 Department of Biology, University of Rome “Tor Vergata”, 00133 Rome, Italy; montesan@uniroma2.it; 8 National Research Council, 00185 Rome, Italy; giulia.cappelli@cnr.it; 9 LAGET, Centre Hospitalo-Universitaire (CHU), N’djamena P.O. Box 456, Chad; 10 EUROBIOPARK and UNSECO Board for Biotechnology, University of Rome “Tor Vergata”, 00133 Rome, Italy; 11 Faculty of Sciences and Technology, Evangelical University of Cameroon, Bandjoun P.O. Box 127, Cameroon; 12 Africa Centres for Disease Control and Prevention, Addis Ababa P.O. Box 3243, Ethiopia; 13 Institute of Human Virology, Baltimore, MD 21201, USA; 14 Bambino Gesù Children Hospital, IRCCS, 00146 Rome, Italy; 15 Faculty of Health Sciences, University of Buea, Buea P.O. Box 63, Cameroon; 16 Central Technical Group, National AIDS Control Committee, Yaoundé P.O. Box 1459, Cameroon

**Keywords:** HIV-1, dual therapies, predictive efficacy, non-nucleosides reverse transcriptase inhibitors, integrase strand transfer inhibitor, Cameroon

## Abstract

Dual therapies (DT) combining integrase strand transfer inhibitors (INSTIs) with second-generation non-nucleoside reverse transcriptase inhibitors (2nd-Gen-NNRTIs) offer new possibilities for HIV treatment to improve adherence. However, drug resistance associated mutations (RAMs) to prior antiretrovirals may jeopardize the efficacy of DT. We herein describe the predicted efficacy of DT combining INSTIs + 2nd-Gen-NNRTI following treatment failure among Cameroonian patients. We genotyped the HIV-1 *pol* gene using Sanger sequencing and assessed acquired RAMs to NNRTIs and INSTIs in patients failing treatment from March 2019 to December 2023. Drug susceptibility was interpreted using Stanford HIVdb v9.5, and statistical analyses were performed using SPSS v22. Of 130 successfully genotyped participants (median age (IQR): 38 (27–46) years; 59.2% female), 92.3% had RAMs to NNRTIs and 1.5% to INSTIs. Prevailing RAMs were Y181C (32.3%) among NNRTIs and R263K (0.7%) among INSTIs. Among 2nd-Gen-NNRTIs, etravirine, doravirine and rilpivirine had 43.85%, 41.54% and 38.46% genotypic sensitivity, respectively. Among INSTIs, we found 97.69% efficacy for dolutegravir/bictegravir, 96.15% for cabotegravir and 92.31% for elvitegravir/raltegravir. The overall predictive efficacy of DT was lower among participants who failed 1st-Gen-NNRTI (*p* < 0.001); with etravirine + dolutegravir/bictegravir combination showing the highest score (43.8%). Conclusively, DT combining INSTIs + 2nd-Gen-NNRTIs might be suboptimal in the context of previous ART failure, especially with NNRTI-based treatment in low- and middle-income countries. The general data clearly indicate that without resistance testing, it is nearly impossible to use long-acting dual therapies in previously failing patients.

## 1. Introduction

Antiretroviral therapy (ART) has significantly reduced AIDS-related morbidity and mortality by slowing down HIV replication to the point where the viral load (VL) in the peripheral blood is undetectable [1,2]. To date, triple therapy (TT) regimens remain the “gold standard” for HIV treatment in resource-limited settings, whereas dual therapies (DT) are among the first choices in the majority of Western guidelines. With this ART strategy, the number of new HIV infections has fallen by more than 60% since the peak in 1995–1997 [1]. However, there are still 39 million people living with HIV (PLHIV) globally (with Sub-Saharan Africa accounting for more than 70%), and 1.3 million people newly infected in 2022 [1]. In 2022, in Cameroon, 480,228 people were living with HIV, with 9898 new HIV infections and 10,198 people dying of an AIDS-related illness [3]. Despite efforts to move towards remission or even lasting eradication of HIV, lifelong ART regimens appear to be the only option for patients to restore and maintain good health [4]. However, in the context of lifelong ART, there is a growing need for treatment simplification strategies to minimize the cumulative toxicity of drug exposure [5], while also improving patients’ adherence to their ART. For more than two decades, in low- and middle-income countries (LMICs) like Cameroon, the backbone for all ART-regimen lines (first, second and third) has been the combination of two nucleoside/nucleotide reverse transcriptase inhibitors (NRTIs) such as tenofovir disoproxil fumarate (TDF), lamivudine (3TC), zidovudine (AZT) or abacavir (ABC) [6]. Of note, some of these NRTIs have been associated with bone, renal and metabolic toxicities that are superimposed on the comorbidities associated with aging [7,8,9] and can lead to significant problems, including long-term toxicity and adherence [10,11]. Therefore, managing PLWH with multimorbidity and polypharmacy remains a significant challenge for clinicians and requires the implementation of new ART strategies like DT. DT combining integrase inhibitors and non-NRTIs (NNRTIs) represents a promising regimen in aging HIV-infected individuals with long exposure to nucleoside analogues and protease inhibitors (PIs) [5]. Etravirine (ETR), rilpivirine (RPV) and doravirine (DOR), the second-generation NNRTIs, are three recently approved NNRTIs by the U.S. FDA (United States Food and Drug Administration) that can be effective against HIV variants resistant to first-generation NNRTIs [12,13,14,15]. Integrase strand transfer inhibitors (INSTIs), i.e., raltegravir (RAL), elvitegravir (EVG), dolutegravir (DTG), bictegravir (BIC) and cabotegravir (CAB), are highly potent, with limited toxicity, and have become the cornerstone of modern ART worldwide [16]. Therefore, to control HIV viral replication, two of these new drugs (INSTIs and 2nd-Gen-NNRTI) are sufficient.

In many studies, these drugs, combined in DT, have shown promising results for ART-experienced patients. For example, DT combining raltegravir with etravirine (ANRS 163 ETRAL trial) [17,18], DTG with RPV (SWORD-1 and SWORD-2 studies) [19,20] and long-acting CAB plus RPV (FLAIR and ATLAS clinical trials) [21,22] maintained a high level of viral suppression over 96 weeks in long-term, experienced HIV-infected individuals. Among INSTIs with 2nd-Gen-NNRTI combinations studied, only the long-acting injectable (LAI) RPV + CAB and RPV + DTG combinations have been validated and approved by the U.S. FDA [12].

In general, DT is a recommended option for individualized treatment of HIV-1 infection in the Western world, with HIV viral load <50 copies/mL on oral ART, no history of virological failure and no history of NNRTIs drug resistance mutations (RAM). In particular, long-acting therapies are now considered of paramount importance for a number of reasons, such as increasing efficacy, reducing the negative effects of lack of adherence and limiting stigma. For all these reasons, long-acting therapies are seen as a relevant option in low- and middle-income countries, especially in Africa. However, in countries, like Cameroon, there is a large proportion of people with previous exposure to/experiencing failure with NNRTI-containing regimens (and archived NNRTI resistance) [23,24,25] and high viral subtype diversity [26,27]. HIV-infected populations generally have limited access to virological monitoring and/or genotypic resistance testing, which is likely to lead to an accumulation of NNRTI resistance [23,24,25]. For all these reasons, the potential relevance of this new approach needs to be considered in practice in light of the overall rate of failures and resistance to INSTI and/or NNRTI, in order to evaluate which patients—how many are they—have real chances to benefit from this approach.

Therefore, the aim of this study was to explore the potential targets eligible for DT among patients experiencing virological failure, in order to optimize the use of DT in our context. Using HIV genotypic resistance testing, we assessed the predictive efficacy of dual ART combinations (approved, in development, not reported to be in development) based on second-generation INSTI and 2nd-Gen-NNRTI among PLHIV failing their ART in Cameroon. Specifically, we analysed genetic variations in HIV-1 *pol* sequences to describe the predicted efficacy of DT combining 2nd-INSTIs + 2nd-Gen-NNRTI following treatment failure in Cameroon.

## 2. Materials and Methods

### 2.1. Study Population and Design

A laboratory-based cross-sectional study was carried out from March 2019 to December 2023, among PLHIV failing their ART (viral load ≥ 1000 HIV-1 RNA copies/mL following an unsuppressed VL and adherence support) who were received for genotypic resistance testing at the Virology Laboratory of the “Chantal BIYA” International Reference Centre for research on HIV/AIDS prevention and management (CIRCB) in Yaoundé-Cameroon. Available demographic data, including age and gender, as well as clinical data such as ART regimen, duration on ART and viremia, were collected from clinical files.

The virology laboratory of CIRCB is a national reference laboratory for HIV-1 genotypic drug resistance testing and a reference for surveillance of HIV drug resistance at the country level (https://www.circb.cm/btc_circb/web/, accessed on 2 April 2024).

### 2.2. Laboratory Methods

#### 2.2.1. Sanger Sequencing Procedure

Viral RNA was extracted from plasma samples using the QIAmp Viral RNA Mini Kit (Qiagen, Hilden, Germany). HIV-1 amplification and sequencing of the approximately 1200 base pair (bp) protease and reverse transcriptase regions were performed using a previously described in-house RT-PR genotyping assay [28]. HIV-1 integrase (864 bp) was amplified and sequenced using a highly sensitive integrase genotyping assay [29]. Capillary electrophoresis was carried out using an Applied Biosystems 3500 genetic analyser (Applied Biosystems, Waltham, MA, USA).

#### 2.2.2. Bioinformatics Analysis

Sequences were assembled and edited using RECall (CDC, Atlanta, GA, USA). Sequence analysis for interpretation was performed using the Stanford HIV database algorithm v9.5 (https://hivdb.stanford.edu/page/algorithm-updates/, accessed on 2 May 2024). All generated sequences were aligned using BioEdit version 7.2.6 (Tom Hall, Raleigh, NC, USA) and compared to reference sequences from the Los Alamos database (https://www.hiv.lanl.gov/content/sequence/BASIC_BLAST/basic_blast.html, accessed on 2 May 2023); HIV subtypes were identified using the Nextclade v3.8.2 sequence analysis web app (https://clades.nextstrain.org/, accessed on 14 June 2023).

### 2.3. Analyses of Potential Drug Susceptibilities

The potential efficacy of dual combinations involving 2nd-Gen-NNRTIs with INSTIs was estimated based on the genotypic susceptibility prediction provided by the Stanford HIV database algorithm v9.5 (https://hivdb.stanford.edu/hivdb/by-patterns/, accessed on 2 May 2024). INSTIs and NNRTIs susceptibilities were interpreted using the genotypic scoring system for drug susceptibility with the following penalty scores: ≥60: high resistance; 15–59: intermediate resistance; <15: susceptible.

### 2.4. Statistical Analysis

Qualitative variables were presented as numbers and percentages, while quantitative variables were presented as median and interquartile range (IQR). For the statistical tests, comparisons were performed using the Chi-square test (χ^2^) or Fisher’s exact test, wherever appropriate for categorical variables, using the Statistical Package for Social Science (SPSS) software version 22. The statistical significance level was set at *p* < 0.05 for all tests.

### 2.5. Ethical Considerations

Administrative authorization was obtained from CIRCB (reference N° 1855/22, issued on 15 September 2022), and ethical clearance was obtained from the Institutional Ethics Committee of the Faculty of Medicine and Biomedical Sciences of the University of Yaoundé I (reference N° 0043/UY1/FMSB/VDRC/DAASR/CSD, approved on 6 February 2023). Unique identifiers and a protected database were used for confidentiality purposes and privacy in data management.

## 3. Results

### 3.1. Characteristics of the Study Population

A total of 130 participants were included in this study. Participants were predominantly female (59.2%), and the median (interquartile range IQR) age was 38 (27–46) years. The median (IQR) ART duration was 84 (42–144) months; 52 (40.0%) participants were failing regimens based on 2 NRTIs + 1 NNRTI, 66 (50.8%) based on 2 NRTIs + 1 ritonavir-boosted protease inhibitor (PI/r) and 12 (9.2%) based on 2 NRTIs + 1 INSTI. Overall, 123 participants (94.6%) had a current/previous exposure to NNRTI-containing regimens, versus only 9.2% exposure to INSTI-based regimens.

The sociodemographic and clinical data of the participants are shown in Table 1.

### 3.2. Subtype Analysis

**Figure 1:**
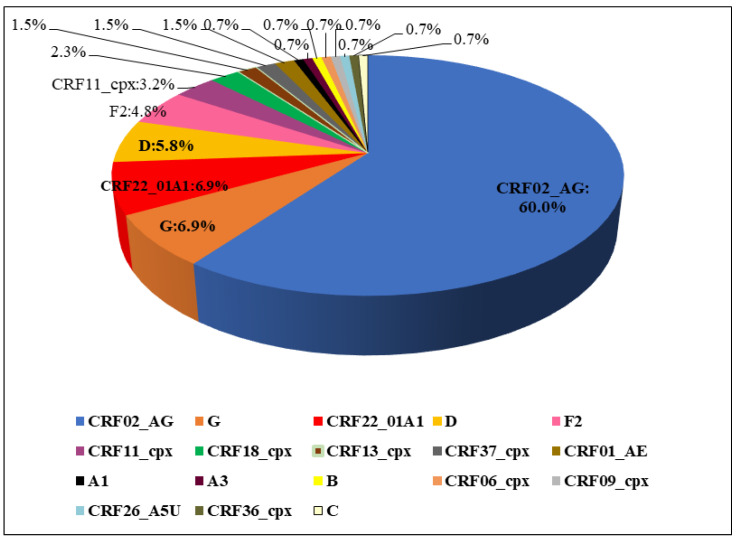
HIV-1 subtype distribution in the study population.

Among the 130 PR/RT sequences, phylogenetic analysis (Figure 1) revealed that CRF02_AG (60.0%; 78/130) was the most prevalent HIV-1 strain, followed by the subtypes G (6.9%), CRF22_01A1 (6.9%), D (5.8%), F2 (4.8%), CRF11_cpx (3.2%) and other HIV-1 subtypes such as CRF18_cpx (2.3%), CRF13_cpx (1.5%), CRF37_cpx (1.5%), CRF01_AE (1.5%), A1 (0.7%), A3 (0.7%), B (0.7%), C (0.7%), CRF06_cpx (0.7%), CRF09_cpx (0.7%), CRF26_A5U (0.7%) and CRF36_cpx (0.7%) were also detected, but with a low frequency.

### 3.3. Genotypic Profiles of the Study Population at Failure

Concerning RAMs to NNRTIs, 120 (92.3%) harboured NNRTI resistance; 96.6% (119/123) in the NNRTI-exposed group and 14.2 % (1/7) in the NNRTI-non-exposed group; *p* < 0.01).

Concerning NNRTIs and specifically 2nd-Gen-NNRTIs (i.e., DOR, ETR and RPV), prevailing RAMs were Y181C (32.3%), V179L (13.8%), K101PE (10.7%) and H221Y (6.9%) (Table 2). Drug susceptibility assessment revealed that 58.5% (76/130) of patients had intermediate to high-level resistance to DOR, 56.2% (73/130) to ETR and 61.5% (80/130) to RPV (see Table 3). Among participants who switched to NNRTI-based regimens (N = 78), only 7.7% (6/78) had no NNRTI-RAMs. The median ART duration (IQR; years) after switching was not significant between patients with NNRTI-RAMs and patients without NNRTI-RAMS (3.5 (2.0–6.0) vs. 6 (5.0–7.5), respectively; *p* = 0.1).

Regarding RAMs to INSTI, two (1.54%) participants harboured INSTI-RAMs. Both cases were found in INSTI-exposed patients (2/12 (16.6%)), and no resistance was observed in the INSTI-non-exposed group. Among participants harbouring major RAMs to INSTIs, we found specifically E138K (1/130), G140A (1/130), Q148R (1/130), R263K (1/130), and S147G (1/130) (Table 2). Some participants harboured a polymorphic INSTI-selected mutation such as T97A (18.5%) and L74I (22.3%).

Stanford genotypic susceptibility revealed that 3.9% (5/130) of participants harboured an intermediate or high level of resistance to CAB and 2.3% (3/130) to DTG/BIC (see Table 3).

Following genotyping, 9/130 (6.8%) of participants did not harbour any RAMs to NNRTIs or INSTIs.

### 3.4. Predicted Efficacy of Dual Therapy Combining INSTIs and 2nd-Gen-NNRTI

**Figure 2:**
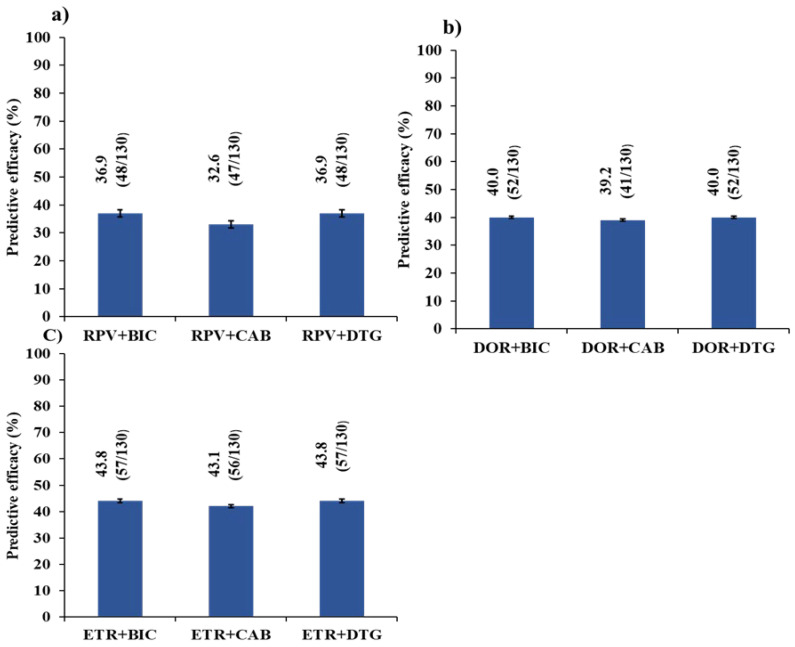
Predictive efficacy of potential DT combining INSTIs and 2nd-Gen-NNRTs. (**a**) Frequency of patients who are susceptible to RPV + INSTIs; (**b**) Frequency of patients who are susceptible to DOR + INSTIs; (**c**) Frequency of patients who are susceptible to ETR + INSTIs. The error bars represent the 95% confidence interval.

Figure 2 summarizes the predictive efficacy according to the Stanford database (scored as <15) of all possible DT combining 2nd-Gen-NNRTIs combined with INSTIs. Overall, the dual regimen with highest predictive efficacy was ETR + INSTIs, whereas the lowest was RPV + INSTIs.

### 3.5. Determinants of INSTIs + NNRTI Efficacies in Dual Therapies

**Figure 3:**
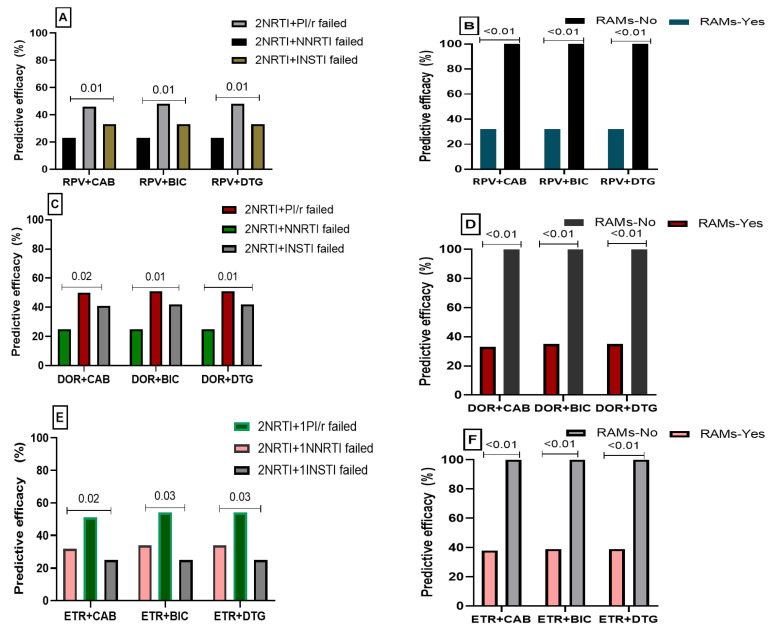
Determinants of the predictive efficacy of a potential dual-therapy-based second-generation NNRTIs + INSTIs. Legend: (**A**) Predictive efficacy of CAB + RPV according to previous treatment failure history; (**B**) Predictive efficacy of CAB + RPV according to the presence of RAMs; (**C**) Predictive efficacy of CAB + DOR according to previous treatment failure history; (**D**) Predictive efficacy of CAB + DOR according to the presence of RAMs; (**E**) Predictive efficacy of CAB + ETR according to previous treatment failure history; (**F**) Predictive efficacy of CAB + ETR according to the presence of RAMs.

Globally, the statistics revealed that age, duration of treatment and viral subtype were not associated with the efficacy of various potential DT. However, the potential efficacy appeared to be lower (*p* < 0.05 in all cases) among participants who previously failed an NNRTI-based protocol and those harbouring RAMs (Figure 3).

## 4. Discussion

Simplified single-pill-a-day tritherapy has radically changed patients’ lives. Currently, there are further strategies aimed at reducing the number of drugs or the frequency with which they are taken, with a good safety profile. INSTIs and 2nd-Gen-NNRTIs are an HIV ART that has demonstrated efficacy in treatment-naive, treatment-experienced and drug-resistant subjects [14,30,31,32]. Studies of INSTIs in combination with 2nd-Gen-NNRTIs provided the most definitive evidence supporting a role for dual therapy [33]. This study evaluated the predictive efficacy of co-administration of INSTIs with 2nd-Gen-NNRTI-based antiretroviral strategies to determine whether these new regimens can be considered suitable alternatives to standard regimens as replacement therapy for patients in virological failure.

In the reverse transcriptase (RT) region of HIV-1 pol, analysis showed a high prevalence of NNRTI-RAMs, including Y181C, Y188C and K101PE, which significantly reduce the efficacy of 2nd-Gen-NNRTIs. This high rate of NNRTI-RAMs can be explained by the fact that about 95% had failed first-generation NNRTIs (EFV and NVP) due to their low genetic barrier to resistance [23,24,25].

However, 43.85%, 41.54% and 38.46% of the study population remained susceptible to etravirine, doravirine and rilpivirine, respectively. In fact, the previous reported mutations, although frequent, do not necessarily affect the efficacy of 2nd-Gen-NNRTIs at the same levels. For example, Y181C does not affect DOR [34].

Concerning integrase inhibitors, major INSTI-RAMs, such as R263K, S147G, G140A and Q148R, were very rare (<2%). Our study differs from that of Ndashimye et al. (2020) in Uganda, who observed a higher rate of INSTI-RAMs in 47% of patients [35]. This disparity could be explained by variations in patient selection and inclusion criteria. In fact, this Ugandan study was carried out in 52 patients failing a RAL-based regimen. Most samples contained minor mutations such as L74I (29/130), which can modulate susceptibility without causing complete resistance [36,37]. One factor that appeared initially to be associated with virologic failure to long-acting CAB + RPV was the L74I integrase polymorphism; however, its role in virologic outcome was unclear [22,38]. According to the Stanford HIVdb algorithm, the predictive efficacy (96.2%) of CAB was lower than that of BIC and DTG (97.7%). This is because DTG and BIC are second-generation INSTIs with a higher genetic barrier to resistance than cabotegravir [39]. Despite the small proportion of patients exposed to INSTIs, these results suggest that INSTIs such as dolutegravir remain a viable option for the management of HIV infection in our context.

Depending on certain factors, this relatively favourable genotypic resistance profile justifies the use of this DT combining INSTIs/2nd-Gen-NNRTIs in the context of virological failure. Overall, the different combinations of INSTIs/2nd-Gen-NNRTIs proved effective, regardless of age, duration of treatment or viral subtype. However, participants who have failed on first-generation NNRTI-based regimens (EFV or NVP) were less sensitive to DT INSTI/NNRTI-based DT compared to those who have failed on PI/r or INSTIs. In fact, virological failure under EFV/NVP-based regimens is often accompanied by the accumulation of NNRTI-RAMs in the HIV genome, which confer cross-resistance to 2nd-Gen-NNRTIs (ETR, DOR, RPV) [40,41]. Indeed, NNRTIs are known to have a low genetic barrier (a single mutation such as M230L is enough to cause resistance to all drugs in the class) [42]. On the other hand, participants in virological failure without mutations to any of the NNRTIs or INSTIs all (100%) showed total efficacy with DT INSTI/2nd-Gen-NNRTIs, underlining the importance of genotypic resistance testing to aid the selection of potential candidates for DT despite exposure to previous ART regimens.

Among the DT- INSTI/2nd-Gen-NNRTI combinations approved by the FDA, only the efficacy of the long-acting injectable combination (LAI) CAB + RPV has been evaluated in Africa through the CARES randomised trial (in Uganda, Kenya and South Africa) [43]. This clinical trial included participants with viremia below 50 copies/mL, who had never failed therapy. In this study, long-acting CAB + RPV therapy had non-inferior efficacy compared with oral therapy, with a good safety profile, and could be considered for African treatment programs [43]. In our study, genotypic analysis predicts the efficacy of CAB/RPV in 36.2% (47/130) of participants after virological failure. This low rate of genotypic susceptibility is mainly due to the high rate of NNRTI-RAMs in our study population. In fact, certain specific mutations present in patients undergoing treatment with efavirenz or nevirapine may also reduce the efficacy of RPV [44]. This high prevalence of NNRTI resistance has previously been described in several studies in our context [25,41]. Consequently, the efficacy of a long-acting injectable protocol may be suboptimal in our context due to the phenomenon of cross-resistance to RPV. The low susceptibility of HIV to DT combining CAB + RPV observed in this study advocates for the use of LAI CAB + RPV only among compliant individuals, with undetectable viremia (<50 copies/mL). In addition, it is worth noting that a careful assessment of NRTI- RAMs among ART-failing individuals may also help to understand the potential efficacy of DTG + 3TC dual therapies alongside that of LAI in these settings. These data also call for further investigation into viral reservoirs in people with virological success (<50 copies/mL), and even in ART-naïve individuals.

## 5. Conclusions

In patients with virological failure, INSTI + 2nd-Gen-NNRTI combinations retain potential efficacy in less than half of the participants, and this is attributable to the very high rate of failure with the emergence of RAMs to 1st-Gen-NNRTI-based regimens. However, the introduction of these DTs must be systematically supported by HIV genotypic resistance testing. Considering the long exposure to 1st-Gen-NNRTIs, it would be prudent to reserve this therapeutic strategy (like the long-acting injectable RPV + CAB protocol) for adherent patients with no history of therapeutic failure to 1st-Gen-NNRTI (EFV or NVP) regimens. The general data clearly indicate that, without resistance testing, it is nearly impossible to use long-acting dual therapies in previously failing patients.

## Figures and Tables

**Table 1:** Baseline characteristics of the participants.

Parameters	Categories or Medians [IQR]	Overall (*N* = 130)
Gender, n (%)	Male	53 (40.8)
	Female	77 (59.2)
Age (years)	Median (IQR)	38 (27–46)
Age groups (years), n (%)	Adolescents	21 (16.2)
	Adults	109 (83.8)
Viremia (at the time of failure, copies/mL)	Median (IQR)	35,078 (5746–178,655)
ART duration (months)	Median (IQR)	84 (42–144)
NNRTI plus 2NRTIs regimens, n (%)	ABC + 3TC + EFV	3 (2.3)
	AZT + 3TC + EFV	2 (1.5)
	TDF + 3TC + EFV	34 (26.2)
	AZT + 3TC + NVP	8 (6.2)
	TDF + 3TC + NVP	5 (3.8)
PI plus 2NRTIs regimens, n (%)	ABC + 3TC + LPV/r	4 (3.1)
	ABC + 3TC + ATV/r	10 (7.7)
	AZT + 3TC + LPV/r	3 (2.3)
	AZT + 3TC + ATVr	6 (4.6)
	TDF + 3TC + LPV/r	3 (2.3)
	TDF + 3TC + ATV/r	40 (30.8)
INSTI plus 2NRTIs regimens, n (%)	ABC + 3TC + DTG	3 (2.3)
	TDF + 3TC + DTG	9 (6.9)

Note: IQR: Interquartile range; 3TC: Lamivudine; ABC: Abacavir; ATV/r: Ritonavir-boosted Atazanavir; AZT: Zidovudine; DTG: Dolutegravir; EFV: Efavirenz; NVP: Nevirapine; LPV/r: Ritonavir-boosted Lopinavir; NNRTI: Non-nucleotide reverse transcriptase inhibitors; NRTI: Nucleotide reverse transcriptase inhibitors; TDF: Tenofovir; PI: Protease inhibitor; INSTI: Integrase strand transfer inhibitor; ART: Antiretroviral therapy.

**Table 2:** Distribution of INSTIs and 2nd-Gen-NNRTI RAMs.

Drug Class	Mutations	Prevalence n (%)
INSTIs *	E138K	1 (0.7)
	G140A	1 (0.7)
	Q148R	1 (0.7)
	R263K	1 (0.7)
	S147G	1 (0.7)
2nd-Gen-NNRTIs	A98G	20 (15.3)
	E138AGKQ	12 (9.2)
	G190ASE	23 (17.6)
	L234I	1 (0.7)
	L100I	4 (3.0)
	K101PE	14 (10.7)
	V179L	18 (13.8)
	Y181C	42 (32.3)
	Y188CL	5 (3.8)
	H221Y	9 (6.9)
	F227FL	8 (6.1)
	M230L	4 (3.0)

* The INSTI mutations were found in two participants.

**Table 3:** Potential efficacies of INSTIs and 2nd-Gen-NNRTIs.

Drug Class	Drugs	Intermediate or High Resistance n (%)	Susceptible n (%)
2nd-Gen-NNRTIs	ETR	73 (56.2)	57 (43.9)
	DOR	76 (58.5)	54 (41.5)
	RPV	80 (61.5)	50 (38.5)
INSTIs	BIC	3 (2.3)	127 (97.7)
	CAB	5 (3.9)	125 (96.2)
	DTG	3 (2.3)	127 (97.7)

Note: ETR: Etravirine; DOR: Doravirine; RPV: Rilpivirine; BIC: Bictegravir; DTG: Dolutegravir; CAB: Cabotegravir; NVP: Nevirapine; LPV/r: Ritonavir-boosted Lopinavir; 2nd-Gen-NNRTI: Second-generation non-nucleotide reverse transcriptase inhibitors; INSTIs: Integrase strand transfer inhibitors.

## Data Availability

Nucleotide sequences for the HIV-1 Pol gene (*RT-PR* and *integrase* region) are available on GenBank under the following accession numbers: OQ985493–OQ985499; OQ985501–OQ985515; OQ985518; OQ985527; OQ985530–OQ985557; OQ985570–OQ985586; OQ985841–OQ985844; OQ985904; OR259498–OR259499; OR365155; OR259747–OR259758; OR259547–OR259558; OR259603–OR259617; OR259672–OR259683; OR259829; MN520217; OK086757; OR365153; MN520219; MW328641–MW328663; MW328665–MW328713; MZ044346–MZ044400.
